# Current Research on the Safety of Pyrethroids Used as Insecticides

**DOI:** 10.3390/medicina54040061

**Published:** 2018-08-28

**Authors:** Agnieszka Chrustek, Iga Hołyńska-Iwan, Inga Dziembowska, Joanna Bogusiewicz, Marcin Wróblewski, Anna Cwynar, Dorota Olszewska-Słonina

**Affiliations:** 1 Department of Pathobiochemistry and Clinical Chemistry, Faculty of Pharmacy, L. Rydygier Collegium Medicum in Bydgoszcz, Nicolaus Copernicus University, 85-094 Torun, Poland; agnieszka.chrustek@gmail.com (A.C.); anna.cwynar@vp.pl (A.C.); dorolsze@cm.umk.pl (D.O.-S.); 2 Department of Pathophysiology, Faculty of Pharmacy, L. Rydygier Collegium Medicum in Bydgoszcz, Nicolaus Copernicus University, 85-094 Torun, Poland; i.dziembowska@cm.umk.pl; 3 Department of Pharmacodynamics and Molecular Pharmacology, Faculty of Pharmacy, L. Rydygier Collegium Medicum in Bydgoszcz, Nicolaus Copernicus University, 85-089 Torun, Poland; joanna.bogusiewicz@gmail.com; 4 Department of Medical Biology and Biochemistry, Faculty of Medicine, L. Rydygier Collegium Medicum of Nicolaus Copernicus University, 85-092 Torun, Poland; marcin.wroblewski@cm.umk.pl

**Keywords:** deltamethrin, permethrin, alpha-cypermethrin, insecticides, human health

## Abstract

Pyrethroids are synthetic derivatives of natural pyrethrins extracted from *Chrysanthemum cinerariaefolium*. They are 2250 times more toxic to insects than to vertebrates due to insects’ smaller size, lower body temperature and more sensitive sodium channels. In particular, three pyrethroid compounds, namely deltamethrin, permethrin, and alpha-cypermethrin, are commonly used as insecticides and are recommended for in-home insect control because they are considered to be relatively non-toxic to humans in all stages of life. However, recent data show that they are not completely harmless to human health as they may enter the body through skin contact, by inhalation and food or water, and absorption level depending on the type of food. Permethrin seems to have an adverse effect on fertility, the immune system, cardiovascular and hepatic metabolism as well as enzymatic activity. Deltamethrin induces inflammation, nephro- and hepatotoxicity and influences the activity of antioxidant enzymes in tissues. Alpha-cypermethrin may impair immunity and act to increase glucose and lipid levels in blood. The aim of the review is to provide comprehensive information on potential hazards associated to human exposure to deltamethrin, permethrin and alpha-cypermethrin. The results of presented studies prove that the insecticides must be used with great caution.

## 1. Introduction

Pyrethroids are synthetic derivatives of natural pyrethrins from the plant *Chrysanthemum cinerariaefolium.* They comprise esters of chrysanthemum acid (ethyl 2,2-dimethyl-3-(1-isobutenyl)cyclopropane-1-carboxylate) and halogenated derivatives of their acids and alcohols [1,2,3]. Natural substances found in the extract of *Chrysanthemum cinerariaefolium* undergo rapid decomposition under the influence of light and therefore they have been replaced with synthetic derivatives, originally considered safe for humans and higher animals [1,2,3,4,5,6].

Pyrethroids belong to the fourth group of insecticides according to the WHO classification and include 42 substances [7]. The mechanism of pyrethroid action is interaction with sodium channels and the induction of prolonged depolarization in neurons. Pyrethroids are divided into two types: Type I and type II, depending on the structure of the compound, and their action and undesirable symptoms they cause. The use of type I pyrethroids may result in the band of tremor type syndrome (T), characterized by tremors of the whole body, aggressive behavior, hypersensitivity and ataxia. The mechanism of action of type I pyrethroids is to change the sodium channels’ conformation during their opening and closing in neuronal membranes [1,2,3]. Type II pyrethroids cause salivation, the choreoathetosis-salivation syndrome (CS) and motor dysfunction in mammals. In addition to their effects on sodium channels, these compounds are known to affect chloride channels, including GABA-dependent ones [1,2,3,8].

Pyrethroids are 2250 times more toxic to insects than to higher animals [1]. This is due to insects having more sensitive sodium channels, a smaller structure and lower body temperature [1]. Moreover, pyrethroids have been shown to be toxic to aquatic animals, e.g., shellfish (crayfish, lobster), and fish (*Danio renio*, carp, rainbow trout), affecting not only the ion channels in neuronal membranes but also in the mitochondrial membranes [4,5,9,10,11,12].

Pyrethroids primarily enter the body through skin contact but also through inhalation or food/water ingestion [13,14,15,16,17,18]. Professional work, water, alimentation and household are the major methods of exposure [7]. It was proved that after the consumption of semolina (pasta), rice, whole meal bread, breakfast cereals and fruits from sprayed areas, the metabolites of pyrethroids were detected in the urine (Table 1) [19,20].

The summary of potential toxic properties of the pyrethroids is presented in Table 1. Pyrethroids, due to their lipophilicity, pass through the blood-brain barrier at concentrations considered to be neurotoxic [2,21,22]. The Center for Disease Control recommends the use of pyrethroid repellents for pregnant women as safe substances to protect against Zika infection [13,23,24], although children are believed to be most vulnerable to pyrethroids [19,21,23].

The aim of the present review is to provide comprehensive information on potential hazards associated with human exposure to three pyrethroids: Deltamethrin, permethrin and α-cypermethrin, which are insecticides recommended for in-home insect control as being relatively non-toxic to humans and mammals. The review contains data on model experiments, animals and human studies published from 1988 to 2018. Due to these pesticides’ degree of toxicity, it is extremely important to investigate the action of pyrethroids, to systematize and publish the available data. Another important issue is that the pesticides are recommended to protect against insect-borne diseases, possibly causing them to be used daily in the household [7,24].

## 2. Permethrin

### 2.1. Chemical Properties and Application

**Figure 1:**
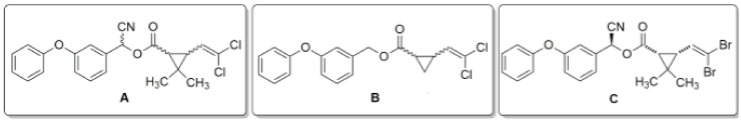
Structure of pyrethroids: (**A**) permethrin—a mixture of *cis*- and *trans*-isomers with a ratio of 1:3, 3-phenoxyphenyl)-methyl] 3-(2,2-dichloroethenyl)cyclopropane-1-carboxylate; (**B**) deltamethrin [(*S*)-Cyano-(3-phenoxyphenyl)-methyl] (1*R*,3*R*)-3-(2,2-dibromoethenyl)-2,2-dimethylcyclopropane-1-carboxylate; (**C**) alpha-cypermethrin—a racemic mixture: [(*S*)-alpha-cyano-(3-phenoxyphenyl)-methyl] (1*R*,3*R*)-3-(2,2-dichlorovinyl)-2,2-dimethylcyclopropane-1-carboxylate oraz [(*R*)-alpha-cyano-(3-phenoxyphenyl)-methyl](1*S*,3*S*)-3-(2,2-dichlorovinyl)-2,2-dimethylcyclopropane-1-carboxylate.

Permethrin belongs to the first group of pyrethroids (Figure 1A). This compound exists in the form of yellow-brown and brown crystals as well as liquid and is soluble in organic solvents [5]. Permethrin occurs in the form of two optical stereoisomers, *cis* and *trans*. Researchers indicate that *cis*-permethrin has stronger neurotoxic properties than *trans*-isomer [21]. Nanometric permethrin with hydrodispersive properties which inhibit the binding of the compound is also used. Consequently, it can be rapidly eliminated from the body because colloidal water dispersion prevents it from aggregating and shortens the retention time in the body [25]. 

Permethrin was registered for the first time in 1979 by the United States Environmental Protection Agency [5,26]. This pesticide is used against mosquitoes and fleas, for the protection of fodder crops and food processing plants, in the construction industry, and in residential areas. It is applied to pets and clothing and it is also used for the treatment of scabies and lice (Table 2) [5,14].

### 2.2. Mechanism of Action

Permethrin may enter the body through the skin, as well as through the gastrointestinal and respiratory tract [5,23]. The LD50 (lethal dose 50%) values of permethrin for acute oral exposure range from 2280–3580 mg/kg, 23.5 mg/L for 4-h inhalation in rats, and 2000 mg/kg after 48 h for dermal exposure in rabbits [5]. Hughes et al. [17,18] demonstrated that up to 20–30% of the pesticide can enter the body of rats through the skin. It has been proven that, depending on the type of food being eaten, the absorption of pyrethroids varies (Table 2) [19,20].

Permethrin causes damage to insect neurons by increasing the impulse conduction, resulting in paralysis and death of insects. The compound disrupts the function of voltage-gated sodium channels: VGSCs in insects and Nav1.6, Nav1.3, Nav1.8 in mammals, delays their inactivation and leads to their premature opening [1,2,3]. To date, research has shown that this pyrethroid exerts its effects on calcium channels by activating the return transport of calcium ions [2,3,29]. It has also been suggested that permethrin may affect the benzodiazepine channels in mammals [1].

Permethrin may cause a variety of symptoms which include epidermal lesions, sore throat, nausea, vomiting, abdominal pain, gastrointestinal mucosal irritation, salivation, respiratory distress and headaches in human [5,6] and animals [1,5,30] upon intoxication. It is metabolized by hydrolysis, esterification, oxidation and conjugation [1,2]. The metabolites of permethrin include *cis*-DCCA (*cis*-3-(2,2-dichlorovinyl)-2,2-dimethylcyclopropane-1-carboxylic acid), *trans*-DCCA (*trans*-3-(2,2-dichlorovinyl)-2,2-dimethylcyclopropane-1-carboxylic acid), 3-PBA (3-phenoxybenzoic acid) (Table 1) [5]. The metabolites are mainly excreted in the urine and faeces. Their half-life is 12.3 h in plasma and 9 to 23 h in neural tissue and the brain [1,31]. It has been shown that the LD50 for rats is 430–4000 mg/kg, for mice 540–2690 mg/kg, and for rabbits over 2000 mg/kg. Permethrin is classified as a non-toxic substance in the case of acute skin exposure and, as for eye irritation, it exhibits low toxicity [5].

### 2.3. Animals Studies

A very important aspect of many studies on pyrethroids is their effect on the reproductive system and fetal progression, as well as after-effects of the exposure to these substances in earlier stages [32,33,34]. The effect of a single dose, orally administered permethrin on pregnancy and fetal growth in animals has been examined [5]. At dose of 150 mg/kg body weight, reduced fetal weight and the presence of additional ribs were observed, while a dose of 50 mg/kg body weight did not cause any adverse effects. In rabbits, a dose of 1200 mg/kg resulted in pregnancy losses and decreased ossification of fore- and hind limbs in fetuses. However, a dose of 600 mg/kg did not produce any undesirable effects [5].

Dohlman et al. [32] suggest that administration of pyrethroids may be detrimental to male fertility, including reduced sperm quality due to endocrine disorders. During the experiment, the influence of 5% solution of permethrin applied topically on dorsum for 19 days on yearling bulls was investigated [32]. The study showed that there was no change in testosterone levels. Additionally, histopathological examination did not show any significant changes. However, a single use of permethrin had slightly adverse effects on sperm morphology [32]. Exposure to pyrethroids at an early stage of gonadal development may harm or delay future spermatogenesis. More immature sperm cells, degenerative changes, and loss of spermatogonia, spermatocytes, Sertoli cells, spermatids and spermatozoa were observed [32].

The effect of permethrin on reproductive organs was also studied [34]. For this purpose, permethrin was administered orally to female rats for 14 days at doses of 20 and 40 mg/kg per day. Electron and light microscopy were used to evaluate rat oocytes. For each dose negative effects on ovarian morphology in a dose-dependent manner were observed, compared with the control group. Pycnotic cells and condensed chromatin were detected, which could lead to apoptosis. In addition, degenerative changes in mitochondria and endoplasmic reticula were found [34].

Omotoso et al. [33] histologically evaluated testes of rats exposed to permethrin at doses of 500 and 1000 mg/kg for 2 weeks. Changes in testis structure, a decreased population of mature sperm cells, and reduced interstitial space were observed, which indicated male fertility dysfunction.

It is believed that the cardiovascular system is susceptible to external factors during pregnancy and after birth. Studies suggest that chronic use of permethrin at a dose of 1/50 LD50 leads to oxidative stress and damages heart cells [35,36]. The effect of early treatment with permethrin on the development of cardiac toxicity in 500-day-old rats was studied [36]. The expression of nuclear factor erythroid 2–related factor 2 (Nrf2) and nuclear factor kappa-light-chain-enhancer of activated B cells (NF-κB) genes, calcium levels, heart surface area and glyceraldehyde 3-phosphate dehydrogenase (EC. 1.2.1.12, GAPDH) reference genes were evaluated. A 1.62-fold increase in the GAPDH mRNA level was reported, while NF-kB mRNA was not significantly altered. A significant decrease in heart surface area was observed. The intracellular calcium influx in the heart increased 4.33 times, compared with the control group. In summary, exposure to orally administrated low-dose pesticides at an early stage of life, from 6 to 21 days, may have long-term consequences, such as cardiac hypertension, elevated calcium levels, and the expression of the Nrf-2 gene at an elderly age [36]. In the same study, decreased plasma membrane fluidity, an increase in plasma cholesterol, interleukin 1 (IL-1), 2 (IL-2), 10 (IL-10) and interferon γ (IFN-γ), and a decrease in plasma albumin concentration were observed [36]. Lowered cholesterol levels, decreased reduced glutathione (GSH) levels and increased glutathione peroxidase (EC 1.11.9.1, GPx) activity in heart mitochondria were detected.

The influence of permethrin at a dose of 1/50 LD50 during the early life of rats was analyzed by measuring its metabolites in the plasma and urine of 500-day-old rats [37]. Differences were found in the concentrations of calcium, sodium, 25(OH)-vitamin D, adrenaline, noradrenaline, nitric oxide, cholesterol and urea in serum. However, in the urine, only sodium concentrations were changed. Lower calcium and higher 25(OH)-vitamin D levels point to decreased calcium absorption, while an increase in NO is associated with hypercholesterolemia and hypoalbuminemia. The study showed that permethrin accelerated the aging process by decreasing the glomerular filtration rate and impairing sodium excretion [37].

Permethrin disrupts immune system function. Female mice were treated with permethrin at one dose of 220–1100 mg/kg body weight [38]. To compare, permethrin NOAEL (No Observable Adverse Effect Level) for mice is 140 mg/kg body weight [26]. A dose of 1100 mg/kg resulted in a 32% inhibition of splenic T-lymphocyte proliferation. Permethrin reduced splenocyte proliferation by 72% and thymic cell proliferation by 52–80%. Moreover, increased apoptosis in CD4^−^8^−^ and CD4^−^8^+^ thymocytes was observed, whereas the CD4^+^8^+^ thymocyte subpopulation was significantly reduced.

Gabbianelli et al. [39] suggested that permethrin affects neutrophils in rats. Permethrin was administrated orally by 0.1 value of LD50 (<43 mg/body weight) for 60 days [26]. It was showed a 33-fold increase in the production of superoxide anion and a 67-fold increase in peroxidase hydrogen peroxide (EC 1.11.1.7) activity, compared to the untreated group, which proves increased oxygen and neutrophil activation. The occurrence of neutrophil apoptosis after an hour of incubation with permethrin or its metabolites was also found [39].

Permethrin exerts a strong action against insects, but it is less toxic to humans and domestic animals because of the lower sensitivity of their sodium channels [21,22]. Domestic animals are often exposed to insecticides, which may adversely affect their health. As such, it is very important to follow the approved dosage. In 11 cats treated with one dose of flea treatment specific for dogs with permethrin contained 45–65%, side effects such as muscle tremors, seizures, agitation, and lack of coordination were observed [30]. During the experiment, four cats died. DeGroot et al. [30] also found permethrin to be toxic. They reported the occurrence of epileptic fits in a 2-year-old cat after application of a permethrin-based flea insecticide.

In the spinal cord neurons, permethrin prolongs depolarization time and impairs neuronal conductance, particularly through alterations in the Nurr1 sodium channel, glutamatergic and dopaminergic transmissions [29,31,40]. Experimental animals exposed to permethrin at dose 25 mg/kg p. o., in fetal life showed an increased number of hypermethylated fragments of DNA in neurons [41].

The dose of 100 mg/mL of nanometric permethrin used in a culture of *Escherichia coli*, *Bacillus subtilis*, *Lycopersicum esculentum*, *Curcumis sativum*, *Zea may* and *Allium cepa* did not show any significant toxicity [25].

### 2.4. Human Studies

The use of permethrin in household is often associated with allergies and asthma, especially in children. A study of 300 children living in Baltimore showed a decrease in the levels of anti-inflammatory IL-10 in plasma in compared to populations without contact with pyrethroid [6]. Goksugur et al. [42] presented a case of a 20-month-old child suffering on scabies treated with 5% permethrin on skin who showed agitation and symptoms such as nausea, vomiting, respiratory distress, tachycardia, and metabolic acidosis. The symptoms subsided after 24 h.

Metabolites of permethrin were detected in breast milk from women in Brazil, Columbia and Spain in concentrations ranging from 1.45 to 24.2 ng/g [43]. Consequently, Glorennec et al. [19] examined the presence of permethrin metabolites in the urine of children aged 6 years living in Brittany (France), and in household dust in homes where domestic insecticides had been used. The metabolites *trans*-DCCA, *cis*-DCCA and 3-PBA were detected in 95, 64 and 63% of the urine samples, respectively [19].

The US EPA (United States Environmental Protection Agency) has established a safe human dose of 0.25 mg/kg body weight per day for both acute and chronic exposure to permethrin [7]. In 1991, the International Agency for Research on Cancer (IARC) classified permethrin as Class 3, regarded as non-carcinogenic to humans [26]. However, recently the US EPA has reclassified it as likely to be carcinogenic to humans by ingestion, based on mouse studies where benign lung and liver tumors were observed [24,26]. 196 women in the second and third trimesters of pregnancy treated with one dose of 4% permethrin used on skin, were screened for effects of the substance on pregnancy outcomes and fetal growth and no adverse effects were found [5].

In humans, long-term exposure of children to permethrin caused an increase in the amount of permethrin metabolites in the urine, as well as behavioral changes, in particular an increase of aggressive behaviors has been observed [44]. Some data also confirmed that consuming food containing permethrin causes problems with short-term memory and concentration [45].

A new concept is the use of the nanometric form of permethrin. Sundaramoorthy et al. [25] evaluated the effects of nano-permethrin at concentrations of 10, 25, 50 and 100 mg/mL on cytotoxicity and genotoxicity in vitro and of permethrin on erythrocytes and lymphocytes by studying erythrocyte morphology and cell viability by means of the cytokinesis-block micronucleus (CBMN) assay. A drastic increase in echinocytes (incorrect erythrocytes) after 24, 48 and 72 h of treatment was observed. Additionally, with increasing concentrations of nano-permethrin and permethrin, a decrease in the viability of human lymphocytes was found [25]. The CBMN test showed a correlation of the number of micronuclei, and trinucleated and tetranucleated cells with different doses of the compounds.

## 3. Deltamethrin

### 3.1. Chemical Properties and Application

Deltamethrin belongs to type II pyrethroids (Figure 1B). This substance is insoluble in water but soluble in alcohol and acetone, and it is lipophilic. It is found in the form of colorless, white and/or light beige crystals [27,46].

Deltamethrin was described for the first time in 1974 and registered by the US EPA in 1994 and is not classified as teratogenic, mutagenic, carcinogenic, or genotoxic [27,46,47].

It is used in crops. It effectively controls aphids, whiteflies, lice, tse-tse flies, fleas, ticks, spiders, ants, bees, bedbugs and cockroaches [31]. It is also effective against vectors of malaria, such as *Aedes aegyptii* and *Anopheles gambiae*, so mosquito nets are often soaked in deltamethrin (Table 2) [24,48].

### 3.2. Mechanism of Action

The neurotoxic activity of deltamethrin is connected with the prolonged opening of voltage-gated sodium channels which results in membrane depolarization of neurons, repetitive discharges and synaptic disturbances leading to hyperexcitatory symptoms of poisoning in insects [2,3]. Deltamethrin also influences the function of the chloride and calcium channels of the neuron [1,3].

Deltamethrin can get into the organism of animals, including mammals, through the digestive, respiratory and skin systems. However for mammalian skin, even short-term contact leads to changes in the transport of ions, mainly sodium [49]. The LD50 values for deltamethrin to acute oral exposure ranges from 30 to 5000 mg/kg, 2.2 mg/L for 4-h inhalation in rats, and 2000 mg/kg after 72 h for dermal exposure in rabbits [46].

In mammals, deltamethrin is metabolized in the liver by ester hydrolysis, oxidation and conjugation with acid residues (glucuronides) and excreted in the urine and faeces (Table 1). The plasma half-life is 10 to 11.5 h, while in urine it is 10 to 13.5 h [27].

### 3.3. Animal Studies

Deltamethrin exhibits low toxic effects on animals. Beagle dogs were tested during an experiment and were fed deltamethrin for 24 months at 1, 10 and 40 μg/g [50]. No adverse effects were observed. Nonetheless, a dose of 10 mg/kg body weight per day led to signs of abnormal movements, convulsions and changes in blood biochemical parameters. It is worth noting that the LD50 for rats is from 30 mg/kg to 5000 mg/kg, for rabbits 2000 mg/kg for skin exposure, for rats from 700 mg/kg to 2940 mg/kg. The respiratory toxicity of deltamethrin in rats is low. The 4-h inhalation LC50 is 2.2 mg/L and the LC50 for a 2-h exposure is 4.6 mg/L [50].

Deltamethrin caused changes in the immune response in rats fed with the substance for 84 days at a dose of 6 mg/kg or 14 days at a dose of 15 mg/kg [6]. Decreased blood lymphocyte activity, splenic plaque-forming cells and roosette-forming lymphocytes were observed. The studies have shown that deltamethrin has a strong affinity for CD45 and CD28 receptors and induces apoptosis in murine splenocytes in a concentration-dependent manner [6,51]. In rats, deltamethrin leads to apoptosis of thymic cells [52]. In summary, deltamethrin is a powerful immunotoxic agent affecting humoral and cellular immunity [53].

In mice, it leads to an increase in the activity of superoxide dismutase (EC 1.15.1.1, SOD) and alanine aminotransferase (EC 2.6.1.2, ALT) [52]. Deltamethrin increases platelets and white blood cell counts. However, it decreases levels of erythrocytes and GPx activity in the blood [52]. Deltamethrin at a single dose of 5 mg/kg, administrated orally induces oxidative stress and activates the mitochondrial pathway of apoptosis by activating caspases [53]. Piperine, a bioactive herbal extract, has been shown to reduce oxidative stress and to affect the activation of caspase-3 caused by deltamethrin [52]. Activated caspase-3 is a death protease which induces apoptosis in spleen cells. The piperine extract has antioxidant, antiapoptotic and chemo-protective properties.

Deltamethrin administered orally at a dose 1.5 and 35 mg/kg, induces hepato- and nephrotoxicity in rats [54]. It was found that deltamethrin causes changes in lipid peroxidation product (malondialdehyde, MDA) levels and decreases antioxidant capacity [54]. Moreover, changes in the activities of SOD and catalase (EC 1.11.1.6, CAT) have been estimated. Increased activity of ALT and aspartate aminotransferase (EC 2.6.1.1, AST), as well as a decrease in GSH concentration have been observed, which may indicate hepatotoxicity [54]. Glutamine protects against hepatotoxicity caused by pyrethroids [54]. The protective effects of spirulina on toxicity in rats have been also observed [55].

Deltamethrin induces nephrotoxicity at a dose 2 mg/kg per day, administered orally for four weeks. Renal damage, changes in serum biochemical parameters, such as urea, uric acid and creatinine, were observed [56]. Deltamethrin reduces acetylcholinesterase (EC 3.1.1.8, ACh) activity in the blood and causes changes in tumor necrosis factor α (TNF-α) levels. It has been shown that ascorbic acid and ceftriaxone may minimize the toxic effects of deltamethrin [56].

The ability to induce inflammation has been investigated by studying cox-2 expression and the ability for apoptosis by analyzing bcl-2 and p53 proteins [57]. An increase in cox-2 and p53 protein levels, and a decrease in bcl-2 levels have been observed, which result in apoptosis. It has been shown that the ethanol extract of olive oil and its phenolic compound, oleuropein, have protective effects on the liver and kidneys of rats treated with deltamethrin by improving the oxidative status and inhibiting inflammation and apoptosis [57]. Oleuropein also has protective, anti-apoptotic effects on neuronal cortical cells treated with deltamethrin administered orally, at a dose of 15 mg/kg for 30 days [58].

Rjeibia et al. [59] have suggested that the methanolic extract from *Amaranthus spinosus* seeds protects hepatocytes treated with 0.6 mg/kg of deltamethrin dose, administered orally. Green tea leaf extract may also exert protective effects against neurotoxicity and oxidative damage caused by the pyrethroid [58].

Deltamethrin affects reproduction and fertility in rats at dose 0.6 mg/kg, injected subcutaneously twice a week for 60 days [60]. In rats fed with deltamethrin, a decrease in the weight of reproductive organs, sperm count, percentage of sperm motility, the amount of sperm and testosterone levels were observed [60]. The study group showed a significant increase in spermatozoa malformations and MDA concentrations. In histopathological studies, disorders of the testicles and epididymis were noted [60]. The experiment showed that supplementation with vitamin E and selenium protected the reproductive systems of rats [60].

Deltamethrin at a dose 10 μM increases plasma Ca^2+^ concentration and induces apoptosis in various tumor cells [61]. This phenomenon has been observed in thymocytes, adrenal cells, the cerebral cortex and hippocampus [51,52,53]. In experimental animals, the intraperitoneal administration of deltamethrin, at a dose 10 mg/kg, influenced BDNF (brain-derived neurotrophic factor)/Tropomyosin receptor kinase B (TrkB) signaling pathways, which are supposed to be the site of the action of drugs used in mental disorders and neurodegenerative diseases [62].

### 3.4. Human Studies

As deltamethrin can be also used as a plant protection product, it is necessary to assess the exposure of persons who work with this pesticide. Hughes et al. [15] investigated the exposure of on the workers of broccoli and maize plantations. Pesticide applicators with similar work experience were dressed in special protective equipment, from which afterward, the pesticide was extracted and evaluated for deltamethrin concentration using a gas chromatography with an electron capture detector. The Potential Dermal Exposure (PDE) was calculated from the concentration of pyrethroid from the extracts of the applicators’ protective clothing. As the authors reported in previous publications, the PDE largely depends on the plant sowing parameters as well as the applicator’s experience [17]. It was observed that higher exposure relates to spraying higher plants—2 m of maize, rather than lower such as broccoli, which was at knee height. The highest PDE was observed for the frontal parts of arms, shoulders and chest. However, the authors suggested that the PDE obtained was relatively safe for the applicator using the deltamethrin spray [15]. It has been estimated that up to 9% of deltamethrin can be absorbed through the human skin in vivo [17,18].

Long-term human exposure to deltamethrin at a dose 0.25–1% through insecticide-soaked mosquito nets may cause headaches, lacrimation, abdominal pain, nausea, diarrhea, vomiting, apathy, weakness, ataxia, limb spasms, convulsions, allergic reactions, hypersensitivity to sound and touch, anaphylactic shock and facial edema [48]. In humans, symptoms of poisoning may occur after taking doses of 2 to 250 mg/kg body weight. The acceptable daily dose is 0.01 mg/kg. Depending on the way of exposure and type of food, the deltamethrin content changes (Table 2).

There are some reports on the effects of deltamethrin on the human body [47,50]. In the case of skin exposure, paresthesias were the most frequently reported effects. Tingling, itching, burning and numbness of the skin may also occur. Severe deltamethrin poisoning was diagnosed in spraymen working in cotton fields [63]. Symptoms such as dizziness and headaches, nausea, fatigue, blurred vision, a burning sensation and tingling of the face, tremor of the arms and legs, convulsions, sun sensitivity and unconsciousness were observed [50,63].

Deltamethrin administered orally or through the skin may accumulate in brain neurons [64,65,66]. Deltamethrin-exposed pregnancy may result in changes in fetal central nervous system [65,66]. Children were characterized by sleep disorders, impaired memory, poorer verbal abilities and decreased intelligence scores [65,67]. Deltamethrin acts on the neuronal dopamine transporter, which may contribute to Parkinson’s disease [67].

## 4. Alpha-Cypermethrin

### 4.1. Chemical Properties and Application

Alpha-cypermethrin, an enantiomeric isomer pair, belongs to type II pyrethroids because of its cyano group (Figure 1C) [8,28,68]. It occurs in the form of crystalline powder and as a dense yellow mass [69]. It exhibits stability in neutral and acidic environments. Due to its lipophilic properties, it is found at higher concentrations in adipose tissue, skin, ovaries, kidneys, adrenal glands, and in the liver [70].

Alpha-cypermethrin is used in growing of cultivated plants. This substance works effectively against butterflies and beetles. It is applied to pets, clothing and mosquito nets (Table 2).

### 4.2. Mechanism of Action

The mechanism of alpha-cypermethrin toxic effect on the nervous system is the disruption of sodium ion transport through the cell membrane. The pyrethroid causes continuous opening of the sodium channel, which leads to the continuous depolarization of the membrane and blocking the generation of action potentials [8].

Alpha-cypermethrin poisoning can cause nausea, vomiting, diarrhea, mucosal irritations, salivation, tearing, motor coordination disorder, chorea, inertia, tremors and clonic seizures [1,70]. The pyrethroid is present in the form of two stereoisomers. The LD50 values for cypermethrin to acute oral exposure range from 150 to 500 mg/kg in rats and 2000 mg/kg after 48 h for dermal exposure in rabbits [68,69].

It is metabolized by cleavage of the ester linkage, followed by conversion to PBA and cyclopropanecarboxylic acid (CPA), which are further metabolized by hydroxylation and glucuronidation (Table 1). Evidence is found in the urine and faeces [8].

### 4.3. Animal Studies

Scientific studies prove alpha-cypermethrin toxicity in animals [9,10,28]. It has moderately high toxicity to rats and mice, with the LD50 value of 80 mg/kg body weight. Rats were fed with a diet containing 25, 100, 200, 400 and 800 mg/kg [68]. At the dose of 400 and 800 mg/kg, an abnormal gait, weight loss and diminished food intake, decreased blood protein levels, and increased urine output were observed. In several cases, the weight of the kidneys and liver increased. In mammals, a dose of 300 mg/kg body weight was shown to cause weight loss, food return, motor dysfunction and tremors [69]. In rabbits, skin irritation, surface necrosis, eye irritation, corneal opacity and damage to the cornea were observed [69].

Animal studies show that cypermethrin at a dose 5 to 20 mg/kg for two weeks can permeate the mother-child barrier, causing mitochondrial and neuronal dysfunction, and affecting communication skills in fetuses [71,72]. Alfa-cypermethrin at a dose 0.02 mg/kg/day of pregnancy may have toxic effects on the development of both the fetus and the parent organism [71]. Changes in organ morphology and blood biochemical indicators have been observed in experimental animals exposed to alpha-cypermethrin for years [71]. Hocine et al. [71] demonstrated weight gain in rats, an increase in plasma glucose and lipid concentrations, rise of AST, ALT and alkaline phosphatase (EC 3.1.3.1, ALP) activities in plasma of pregnant rats and their neonates following exposure to alpha-cypermethrin at a dose of 0.02 mg/kg body mass per day.

Alpha-cypermethrin induces apoptosis of cells. Romero et al. [73] performed cytotoxicity assays on SH-SY5Y line cells treated with the substance. They used a MTT colorimetric assay (3-(4,5-dimethylthiazol-2-yl]-2,5-diphenyltetrazolium bromide) and examined lactate dehydrogenase activity (EC 1.1.1.27, LDH). Determination of cytotoxicity showed that alpha-cypermethrin at a dose of 0.01 to 30 μM did not affect SH-SY5Y cells. In contrast, the use of pyrethroid at a dose of 60 μM slightly influenced the viability of cells, as tested by the MTT test, and the occurrence of LDH in the culture fluid. Alfa-cypermethrin at a concentration of 1 to 100 μM resulted in an increase of MDA level [73]. The expression of cell death pathway genes was also studied [71]. There were changes in mRNA levels of 39 genes, including: ATP6V1G2, BCL2, CASP9, FAS, GADD45A, SPATA2, SYCP2, ATG7, NFκB1, SNCA, ULK1 and JPH3. The results indicate DNA damage, which may lead to apoptosis, autophagy and necrosis. 

### 4.4. Human Studies

It has been shown that alpha-cypermethrin affects the human body, particularly in the case of long-term exposure. In the study performed on production workers in Durban, South Africa, an analysis of its metabolites in the worker’s urine was performed [74]. Alfa-cypermethrin metabolites were detected in the urine of people working in cotton fields [16]. Skin lesions on the face and neck were observed. However, the metabolites of the examined pyrethroid were not found in the urine. The pyrethroid concentrations have been analyzed in samples of breast milk of women from Brazil, Colombia and Spain, countries where pesticides are widely used in agriculture and where contracting malaria can occur. The measured concentrations of cypermethrin metabolites have not exceeded accepted standards and ranged from 1.45 to 24.2 ng/g [43].

No mutagenic, carcinogenic, embryotoxic or teratogenic effects were demonstrated, and no reproductive toxicity was observed [8]. Studies suggest that pyrethroids may impair immunity [6,70]. Thirty people working in a greenhouse and exposed to alpha-cypermethrin were tested [70]. There was a decrease in cytokine concentrations, such as IL-2, IL-8, IL-12 and IFN-gamma, which may suggest that changes occurred in anti-inflammatory and anti-cancer responses.

Singleton et al. [16] assessed worker’s exposure to alpha-cypermethrin on the cotton fields in the Nile Delta in Egypt. The 3-PBA and *cis*-DCCA were measured in the employees’ urine samples. The assay was performed at the State University of New York at Buffalo, NY, USA using a gas chromathography with mass selective detector (GC-MSD). The study revealed that the highest levels of cypermethrin metabolites were found in daytime urine samples from applicators [16].

Research among workers exposed to pesticides is very important. Two-hundred workers exposed to alpha-cypermethrin from an Egyptian company were tested [68]. The study subjects were divided according to the degree of exposure, into three groups: Workers most vulnerable to exposure to alpha-cypermethrin (weighing and production departments), workers with moderate risk to alpha-cypermethrin exposure (packing and storage), and employers not exposed to alpha-cypermethrin (administrative department). All subjects were examined for genotoxic and immunological changes. The presence of CD3, CD4 and CD8 immunofenotypes were estimated during the study to evaluate the function of the immune system. CD4 molecules are characteristic for auxiliary T cells, CD8 for cytotoxic T cells, the CD4/CD8 ratio describes the function of the immune system, and CD3 differentiates lymphocytes from other cells that have CD4 and CD8 on their surface. The study revealed that the highest risk for pesticide exposure related to a significantly lower CD4/CD8 concentration. The mutation in the TP53 gene for the suppressor protein was also evaluated. Single stranded conformational polymorphism (SSCP) staining demonstrated changes in exones of this gene in highly and moderately exposed workers [68].

In addition to reduced immunity and mutations within the suppressor gene, oxidative stress can also have a significant effect on functional cells. El Okda et al. [68] demonstrated changes in the concentration of the following selected enzymes involved in antioxidant protection: SOD, CAT, GPx, as well as GSH concentration. The activity of these enzymes, as well as the GSH concentrations were reduced in workers exposed to pyrethroid compared to non-exposed individuals [68].

Studies indicate that immune disorders, gene mutations and oxidative stress occur during exposure to the pyrethroid. Immunotoxicity was analyzed by examining 30 workers exposed to alpha-cypermethrin [68]. These individuals did not show clinical signs or changes in leukocyte subpopulations. However, differences in IL-12, p70, IL-2, IL-8 and IFN-gamma were noted. The results are of great importance because of the role of proinflammatory cytokines in infections and in cancer.

As alpha-cypermethrin affects the central nervous system, prolonged exposure to this substance induced problems with motor coordination and learning, however—in contrast to permethrin exposure—aggressive behaviors have not been observed [75]. Glorennec et al. [19] detected the presence of cypermethrin metabolites in the urine of children living in France. A case of a 45-year-old man who died after eating vegetables with a 10% cypermethrin solution, instead of oil, was also described [76].

## 5. Concluding Remarks

Due to the toxic effects of pesticides on human and animal organisms as well as on the environment, their use is governed by global organizations, such as the World Health Organization (WHO) and the European Union (EU), and, additionally, by the internal regulations of several countries. Only three natural pesticide derivatives of the pyrethroid group are allowed to be used by the WHO, namely, deltamethrin, permethrin and alpha-cypermethrin [7,24,26,27,28,77]. Pyrethroids are insecticides recommended for protection against insects transmitting pathogenic microorganisms to humans and animals. They are widely used not only in agriculture, households, forestry and horticulture, but also in medicine and veterinary medicine [1,2,3,19,28]. Due to the growing threat posed by pathogenic microorganisms, such as *Plasmodium* spp. and the Zicka virus, known to be transmitted by animal vectors, the use of insect repellents and insecticides seems essential to modern society [13,14,23,24,49].

Pyrethroids are characterized by low toxicity to humans [26,27,28]. In adults they are rapidly metabolized, do not accumulate in tissues and are excreted in the urine. They may enter the body through inhalation, food, water, and skin contact [1,2,3,15,17,18]. Data about poisoning with these compounds and about fatalities following their use is regularly published. Children are the most vulnerable risk group, as pyrethroids are most likely to be present in breast milk and, consequently, breastfeeding may be the starting point of their accumulation in tissue [19,23,41,43].

A multiple regression analysis suggests that diet plays an important role in the presence of pyrethroid metabolites, as some dietary products may contain pyrethroids [19]. Thus, identification of these products helps to avoid pyrethroid intoxication. In insects, pyrethroid action consists on the opening of the neuronal sodium channel, which causes a constant depolarization phase [1,2,8]. However, pyrethroid resistance has been reported, consisting of modifying post-translational folding of the channel subunits, which results in different spatial conformation, insensitive to commonly used pyrethroid products [1,3]. In the 1980s, 573 cases of acute pyrethroid poisoning were reported, resulting in the deaths of 7 people [1]. Oral exposure may cause dizziness and headaches, anorexia, fatigue, nausea, vomiting, pulmonary oedema, convulsions, coma and even death [1,15,74].

The issue raised above highlights the importance of tracking reports of poisoning from insect repellents and insecticides as such products are considered safe for use by people of all ages, including pregnant women and infants [13,23,49,67]. So far, the search for insecticides that are toxic to insects and safer for humans has been unsuccessful. The presented studies show that these products are far from harmless to human health, and that every insecticide must be used with great caution. As these are commonly used products that are labeled safe for human use, thorough studies highlighting the long-term physical, neurodevelopmental, neurobehavioral, reproductive and cancer related dangers these pyrethroids pose to both low and high risk (high users) population groups are needed [1,8,22,40,44,65].

## Figures and Tables

**Table 1:** Characteristics of pyrethroids recommended by WHO to be used in the prevention of insect-borne disease [7,10,19,20].

Pyrethroid/Group	Half-Life in the Body	Excreted Substances	Toxic Dose for Humans	Quantity of Active Substance	Negative Effects in Humans
Permethrin I (T)	12.3 h in plasma 9–23 in neuronal tissue	*cis*-DCCA *trans*- DCCA 3-PBA	<0.25 mg/kg/day (RfD) >50 mg/kg	3.75–7.5%	Reproductive system Skeletal system Cardiovascular system Immune system Neuronal system
Deltamethrin II (CS)	11.5 h in plasma	3-PBA *cis*-DBCA	<0.01 mg/kg/day (RfD) >30 mg/kg	0.25–0.5%	Immune system Reproductive system Neuronal system Skeletal muscles Inducing oxidative stress
α-cypermethrin II (CS)	2.5 days in fat tissue	F-PBA CPA	<0.05 mg/kg/day (RfD) >50 mg/kg	0.25–0.5%	Immune system Reproductive system Neuronal system Inducing apoptosis in SH-SY5Y line cells

CS—choreoathetosis-salivation, T—tremor, RfD—reference dose, 3-phenoxybenzoic acid (3-PBA), 4-fluoro-3-phenoxybenzoic acid (F-PBA), *cis*-3-(2,2-dichlorovinyl)-2,2-dimethylcyclopropane-1-carboxylic acid (*cis*-DCCA), *trans*-3-(2,2-dichlorovinyl)-2,2-dimethylcyclopropane-1-carboxylic acid (trans-DCCA), *cis*-3-(2,2-dibromovinyl)-2,2-dimethylcyclopropane-1-carboxylic acid (*cis*-DBCA), cyclopropanecarboxylic acid (CPA).

**Table 2:** Routes of exposure to permethrin, deltamethrin and α-cypermethrin [7,19,20,26,27,28]

Pyrethroid/Group	Routes of Exposure	Sources (Pyrethroid Concentration)
Permethrin I (T)	Skin	Topical creams for scabies (5%). Flea/tick medicines for pets (25–75%)
	Respiratory tract	Mosquito nets (0.25–1%)
Deltamethrin II (CS)	Skin	The highest accumulation should be expected in: cotton, coffee, hops, artichokes, corn, broccoli, apples, plums and grain (1–2.5%) Flea/tick medicines for pets (0.25–10%)
	Gastrointestinal tract	The highest accumulation should be expected in hops, artichokes, broccoli and grain
	Respiratory tract	Mosquito nets (0.25–1%)
α-cypermethrin II (CS)	Skin	Crop protection chemicals—cotton, rice, potatoes, citrus fruits, grapes and soy (1–2.5%) Flea/tick medicines for pets (3–5%)
	Gastrointestinal tract	The highest accumulation should be expected in soy, citrus fruits and grapes
	Respiratory tract	Mosquito nets (6%)
